# Multi-trait multi-locus SEM model discriminates SNPs of different effects

**DOI:** 10.1186/s12864-020-06833-2

**Published:** 2020-07-28

**Authors:** Anna A. Igolkina, Georgy Meshcheryakov, Maria V. Gretsova, Sergey V. Nuzhdin, Maria G. Samsonova

**Affiliations:** 1 Peter the Great Saint-Petersburg Polytechnic University, Russian Federation, Polytechnicheskaya, 29, St. Petersburg, 195251 Russia; 2 Centre for Genome Bioinformatics, St. Petersburg State University, St. Petersburg, 199034 Russia; 3 Program Molecular & Computational Biology, Dornsife College of Letters Arts and Science, University of Southern California, Los Angeles, CA USA

**Keywords:** GWAS, SEM, Multi-trait multi-locus SEM, Bayesian inference, Chickpea

## Abstract

**Background:**

There is a plethora of methods for genome-wide association studies. However, only a few of them may be classified as multi-trait and multi-locus, i.e. consider the influence of multiple genetic variants to several correlated phenotypes.

**Results:**

We propose a multi-trait multi-locus model which employs structural equation modeling (SEM) to describe complex associations between SNPs and traits - **m**ulti-**t**rait **m**ulti-**l**ocus **SEM** (mtmlSEM). The structure of our model makes it possible to discriminate pleiotropic and single-trait SNPs of direct and indirect effect. We also propose an automatic procedure to construct the model using factor analysis and the maximum likelihood method. For estimating a large number of parameters in the model, we performed Bayesian inference and implemented Gibbs sampling. An important feature of the model is that it correctly copes with non-normally distributed variables, such as some traits and variants.

**Conclusions:**

We applied the model to Vavilov’s collection of 404 chickpea (*Cicer arietinum L.)* accessions with 20-fold cross-validation. We analyzed 16 phenotypic traits which we organized into five groups and found around 230 SNPs associated with traits, 60 of which were of pleiotropic effect. The model demonstrated high accuracy in predicting trait values.

## Background

Understanding how genetic variation translates into phenotypic effects is one of the central challenges facing fundamental biology, agriculture, and medicine. Solutions of this problem fall into two main classes: association studies and trait prediction studies. Genome-wide association studies (GWAS) are designed to identify genetic variants associated with a trait. Initially, GWAS was conducted for each trait separately testing SNPs one by one. However, single-locus approaches may lead to biased estimates due to multiple testing correction, and they are not suitable in the common case of genetically correlated traits.

To alleviate the latter challenge, multi-trait models have been proposed [1, 2]. One way to cope with correlated traits is to model the inter-trait covariance as a random effect in linear mixed effects models [3]. Until recently, this model could use only a pair of correlated traits at a time due to the computational intensity [4]. To avoid this complexity, variable reduction techniques were suggested to replace several phenotypic traits with new independent constructs. These constructs play the role of new traits and can be obtained with a standard principal component analysis of traits (PCA), various principal components of heritability (PCH) [5–7] or pseudo-principal components [8]; however, the biological interpretation of these artificial traits is not clear. Moreover, these methods do not distinguish trait-specific and pleiotropic variants. To carry this out, meta-analysis combining several single-trait GWAS of different traits was proposed [9]. It can derive trait-specific variants, but, as correlated traits were not analyzed simultaneously, this method is not multi-trait by definition.

Another challenge in association studies is to develop a powerful multi-locus model. Single-locus models require correction for multiple testing, which dramatically reduces power. To avoid this problem, multi-locus models that consider all markers simultaneously have been proposed. Due to the ‘large p (number of SNPs), small n (sample size)’ problem, many multi-locus models are based on regularization/penalized techniques: LASSO [10], Elastic Net [11], Bayesian LASSO [12], adaptive mixed LASSO [13]. Other multi-locus methods, which are incorporated in the mrMLM package, involve a two-step algorithm which first selects candidate variants from a single-locus design and then examines them together in a multi-locus manner [14]. Despite their diversity, the multi-locus models are limited in multi-trait cases and seldom pay attention to different types of SNP effects (e.g. pleiotropic, single-trait, direct, indirect).

In contrast to GWAS, the second broad class of studies make genome-wide trait predictions. These studies have gained popularity and enjoy practical application in agriculture, specifically, in estimating individual breeding values and selecting breeding lines [15]. Genomic prediction methods not only search for trait-variant associations but also validate them by demonstrating their predictive ability. Similar to GWAS, these methods are based on various regression models that typically include multiple loci and consider kin relationships between individuals. The latter is usually treated as the random effect, i.e. the multivariate normally distributed variable with zero mean and a covariance matrix proportional to pedigree-based or marker-based kinship [16]. The random effect can be estimated together with marker effects as in BLUP and various GWAS mixed-models [17–19] or before the association analysis as in GRAMMAR [20].

Despite the broad spectrum of multi-trait and multi-locus models in GWAS and trait prediction studies, only a few of them simultaneously incorporate correlated traits and several associated variants [21–25]. In principle, multi-trait and multi-locus models have the potential to reveal complex and important types of associations; for instance, a single variant might have a direct effect on one trait and an indirect impact on the other trait, may act on a single trait or its effect might be pleiotropic affecting several traits. However, none of these traits-variants associations are explicitly embedded into known models. This is why it is tempting to have these relationships described explicitly, as in structural equation models.

Structural equation modeling (SEM) is a multivariate statistical analysis technique first introduced for path analysis by geneticist Sewell Wright [26, 27]. Once predominantly used in genetics, econometric, and sociology, SEM applications have gradually shifted to the field of molecular biology [28]. For example, SEM has been used to explore alterations in gene networks in diseases [29, 30], to provide a quantitative map of relationships between traits and disease [31], and to infer gene regulatory networks involving several hundred genes and eQTLs [32, 33].

SEM models have also been applied in association studies in both multi-trait and multi-locus designs. For example, the GW-SEM method has been developed to test the association of a SNP with multiple phenotypes through a latent construct [34]. In comparison with the existing multi-trait single-locus GWAS software package GEMMA (Zhou and Stephens 2014), GW-SEM provides more accurate estimates of associations; however, GEMMA is almost three times faster than GW-SEM. Another SEM-based model which can be used in association studies has been proposed for multi-trait QTL mapping [35]. This method assumes that phenotypes are causally related forming a core structure without latent constructs, and QTLs play the role of exogenous variable to the structure. This approach allows the model to decompose QTL effects into direct, indirect, and total effects. However, the assumption of causally related traits is limiting because the correlation between traits can additionally be caused by pleiotropy rather than the direct influence of traits on each other. Therefore, the current SEM-based models for genotype-phenotype associations can be improved to address these drawbacks.

Here, we propose a new multi-trait multi-locus SEM-based model – **mtmvSEM** – that considers both correlated traits joined into latent constructs, which can be causally related to each other, and multiple SNPs influencing both traits and latent variables. In contrast to PCA-based approaches, our model does not operate with artificial phenotypes in the form of linear combinations of traits, but rather the phenotypes are regressed on the latent constructs. The proposed configuration of the model distinguishes pleiotropic and single-trait effects of SNPs on latent variables and phenotypes, respectively. Moreover, SNP effects can be differentiated between direct and indirect. This explicit separation of SNP roles may provide a better understanding of genetic mechanisms underlying a trait than other multi-trait multi-locus models.

Our approach faces several challenges. First, in case of a large number of traits and variants, the model potentially belongs to the “large p, small n” class, so that the standard maximum likelihood (ML) method for estimating parameters in SEM models is limited due to the parameter identification criteria. This problem can be solved by applying the Bayesian approach, which uses prior information about model parameters. Bayesian multiple-regression methods are widely used for genomic prediction in agriculture and in GWAS [36] reducing the number of tests, and consequently, increasing robustness and power as compared to standard GWAS analyses [37]. In our model, we performed Bayesian inference and obtained posterior distributions of parameters by Gibbs sampling, a Markov chain Monte Carlo (MCMC) algorithm.

Another challenge in our model is the inclusion of both continuous and ordinal variables given that variants and many phenotypes are measured on ordinal scales. As a result, it is impossible to estimate parameters in SEM models using statistical models relying on the normality assumption. These limitations explain the sparsity of studies conducting SEM analyses in a genome-wide context. In our model, we incorporated techniques to cope with ordinal data – polychoric and polyserial correlations – that provide a correct analysis of genetic variants and traits.

Our model was applied to a dataset of 404 chickpea landraces analyzed recently [38]. Chickpea is the second most widely grown food legume, providing a vital source of nutritional nitrogen for ~ 15% of the world’s population. To accelerate chickpea breeding, it is important to identify regions controlling agronomically important traits. However, while performing GWAS, we found that 16 out of 30 phenotypic traits considered were correlated. Therefore, to obtain statistically reliable markers and to understand the causal relationships between traits and variants, the mtmlSEM model developed here was applied to this dataset. We also used the model to predict chickpea phenotypic traits and got sufficiently good results for most of them.

## Results

### Application of mtmlSEM model to chickpea dataset

**Fig. 1:**
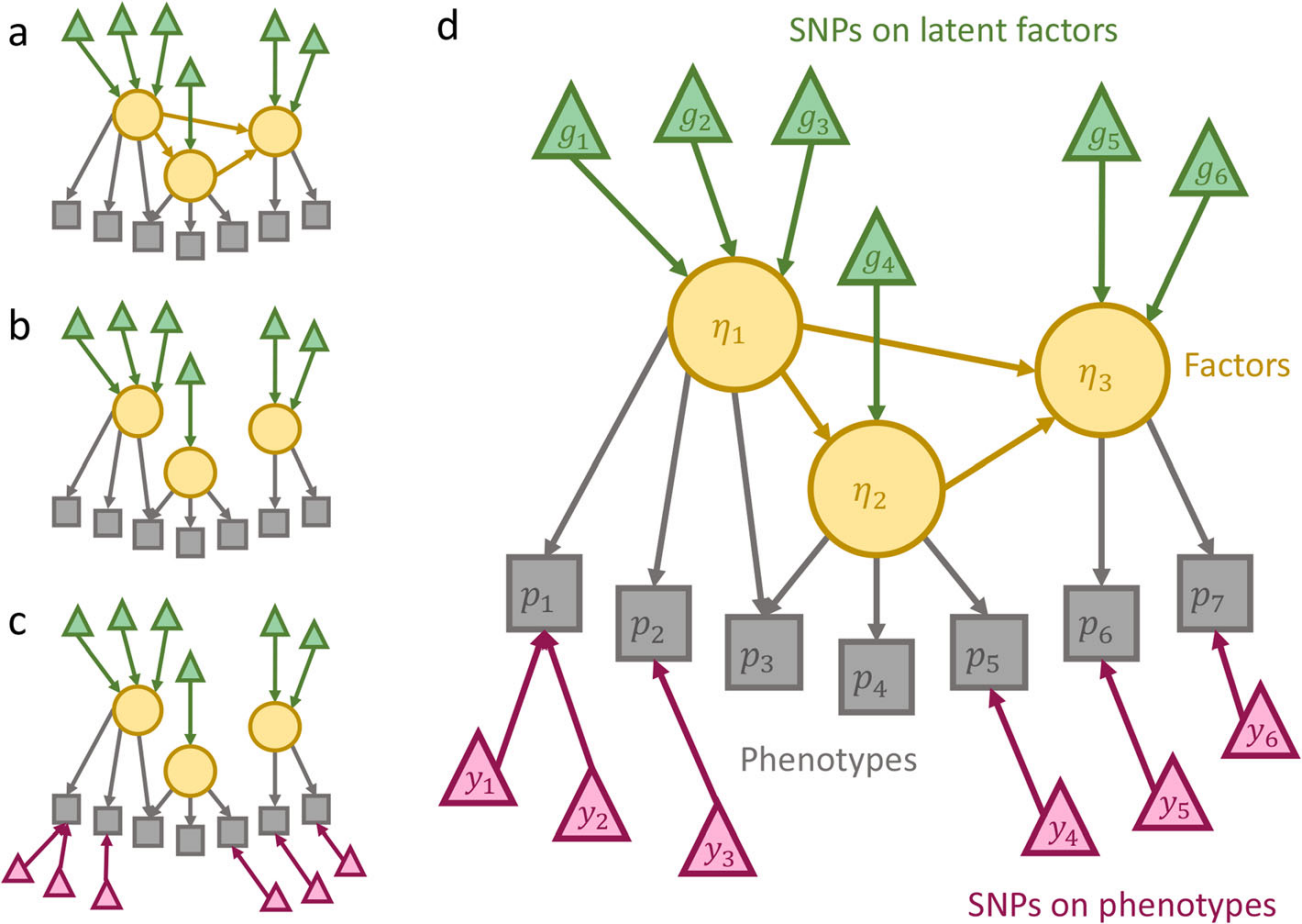
Examples of the genome-wide multi-trait SEM model. **a** Connected base model; (**b**) Zero base model; (**c**) Zero extended model; (**d**) Connected extended model

To test whether the relations between latent factors in the model are reasonable and to evaluate impacts of different types of SNPs, we compared four types of models (Fig. 1). We denote a model having parameters in the B matrix as *connected* and a model without a B matrix as *zero*. We denote a model without the K matrix as *base* and a model having parameters in the K matrix as *extended*. Four model configurations were considered covering all possible combinations (Fig. 1).

For each of the four models, we assessed its predictive ability with the fixed 20-fold cross-validation. In each of the 20 training sets, we automatically obtained the same set of 5 factors influencing 16 partly correlated phenotypes (Table 1, Additional File 1). The first two factors reflect different types of productivity traits. The third factor reflects joint variation in the color of different plant parts. The fourth can be interpreted as a phenological factor. The fifth reflects joint variation of traits related to plant architecture, in particular, plant height and height of the lover pod attachment.

**Table 1:** 5 factors influencing 16 partly correlated phenotypes

Factor	Attributed phenotypes	Description
1	NoPodsWeight	Plant weight without pods
	PodsWeight	Pods weigth
	PodsNumber	Number of pods per plant
	SeedsNumber	Number of seeds per plant
	SeedsWeight	Seeds weight per plant
2	PodLength	Pod length
	PodWidth	Pod width
	Seed1000W	Thousand seeds weight
3	FloCol	Flower colour
	StemCol	Stem colour
	FlowStemCol	Peduncle colour
	SeedCol	Seed colour
4	BegFEndF	Days from beginning of flowering to end of flowering
	EndFBegM	Days from end of flowering to beginning of maturation
5	Height	Plant height
	Hlp	Height of lower pod attachment

**Fig. 2:**
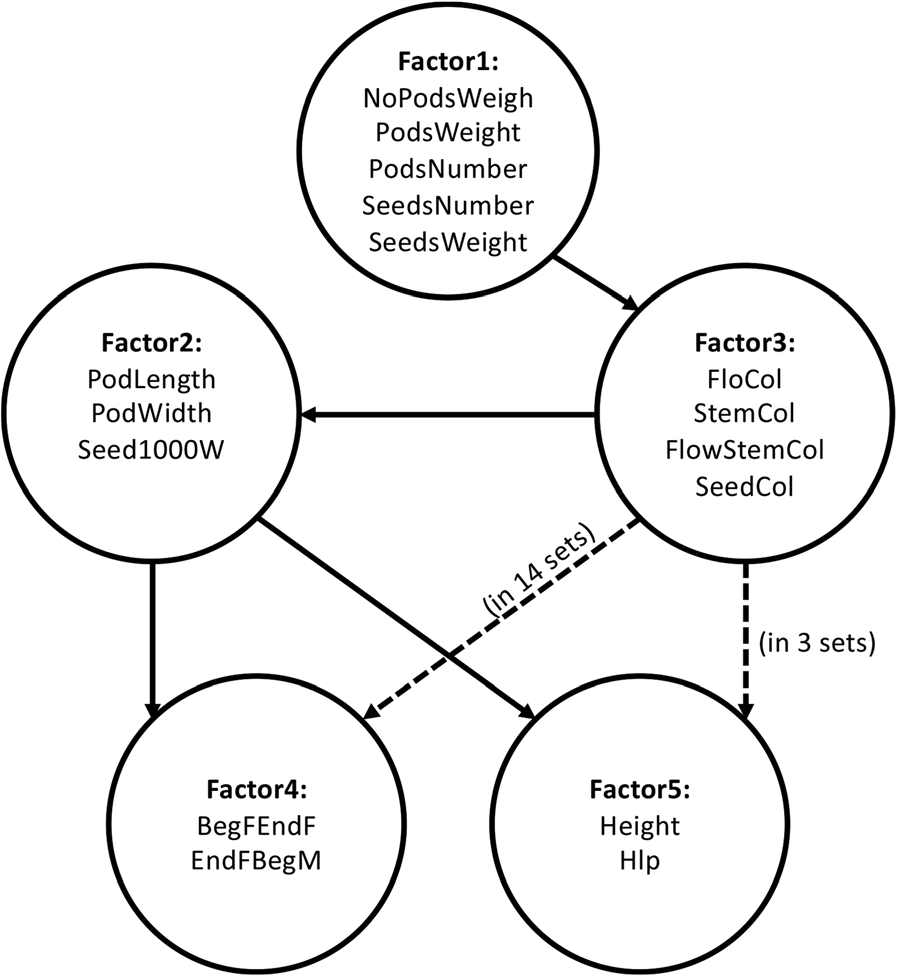
Latent factors joined to form structural part of connected SEM model. Dashed arrows represent relationships, which were not present is all training sets for directed acyclic graph obtained; Solid lanes represent relationships, which were found in each of 20 training sets

In the connected model, the latent factors were joined into a directed acyclic graph and this procedure resulted in slightly different structural parts for the 20 training set models. We found that the number of connections between latent variables varied from four to six with four being common to all training sets (Fig. 2). From the statistical viewpoint, relationships between latent variables reflect their common variances that maximize the likelihood of the sample covariance matrix subject to parameters of the model. However, a biological interpretation of the connections may be that the relationships between factors related to productivity and plant color reflect selection on market class: desi chickpeas have a small dark seed, while kabuli have large lightly colored seeds [39]. Relations between productivity and phenology as well as between productivity and plant architecture are also apparent: plant productivity reflects the efficiency of plant metabolism that obviously influences plant architecture and phenology [40].

We first added SNPs influencing the latent factors to obtain both the connected and zero base models. The number of SNPs in the connected base models constructed for 20 training sets varied from 52 to 62; for zero base models, this number was in the range from 36 to 46. The larger number of SNPs in connected models as compared with zero models can be explained by the essential difference between SNPs attributed to these model types. In connected base models, some SNPs are associated with several latent factors and therefore affect a larger number of phenotypic traits than in zero models. Therefore, in connected models, SNPs describe a more complex variance-covariance structure and, as a result, a larger number of SNPs is required to estimate it.

Notably, SNPs influencing latent factors do not explain the variances specific to individual phenotypic traits. To take into account these variances, we built extended models for each training set. The number of SNPs in connected extended models varied from 223 to 256; in zero extended models, this number was in the range from 218 to 242. The significant increase in the number of SNPs in extended models as compared with base models can be explained by the fact that extended models additionally consider around ten SNPs per each of the 16 traits on average.

To obtain parameter estimates for each of the 80 models (4 model types and 20 training sets), we performed five Gibbs sampling chains of length 2000 and checked several diagnostics with tools in the *coda* CRAN package. The Gelman-Rubin diagnostics was higher than 1.05 in only 1% of all parameters. The minimum effective sample size for a parameter was 83 and the mean and median effective sample sizes across all parameters and models were 3193 and 3304, respectively. Based on these diagnostic values, we concluded that there was good convergence of the Gibbs sampling chains and took parameter estimates for testing.

For all model types, the accuracy of trait prediction is good for plant height, some traits related to productivity, and all traits related to plant color (Table 2, Additional File 2). Closer inspection of the table showed that the connected base model outperformed the zero base model for 9 phenotypic traits, the opposite situation was observed for 5 traits, and predictions for the remaining 2 traits were nearly equal. When comparing the connected and zero extended models, the number of times one model outperforms the other is nearly equal (Table 2) and the number of predictions with equal accuracy increases pointing to greater similarity between these models.

**Table 2:** Accuracy of trait prediction for four models (Pearson correlation between actual values and predicted and coefficient of determination). Bold font: connected model outperforms zero model; Italic font - prediction accuracies of connected and zero models are nearly equal

Model type	Connected		Zero		Connected		Zero	
SNPs influence	extended		extended		base		base	
Measure	corr, r	r^2^	corr, r	r^2^	corr, r	r^2^	corr, r	r^2^
Seed1000W	*0.75*	*0.56*	*0.75*	*0.56*	*0.75*	*0.56*	*0.75*	*0.57*
FloCol	0.69	0.48	0.71	0.50	**0.65**	**0.42**	**0.64**	**0.42**
FlowStemCol	**0.68**	**0.46**	**0.67**	**0.45**	**0.67**	**0.45**	**0.66**	**0.44**
SeedCol	0.67	0.45	0.68	0.46	0.60	0.36	0.63	0.39
NoPodsWeight	*0.67*	*0.45*	*0.67*	*0.45*	**0.59**	**0.34**	**0.57**	**0.33**
PodLength	0.67	0.44	0.69	0.47	**0.65**	**0.42**	**0.64**	**0.41**
StemCol	0.64	0.42	0.67	0.45	0.65	0.43	0.67	0.45
PodWidth	0.63	0.40	0.66	0.44	0.64	0.40	0.67	0.44
Height	*0.59*	*0.34*	*0.59*	*0.35*	**0.57**	**0.33**	**0.49**	**0.24**
PodsWeight	**0.45**	**0.20**	**0.42**	**0.18**	**0.44**	**0.19**	**0.42**	**0.18**
SeedsWeight	**0.38**	**0.14**	**0.36**	**0.13**	**0.38**	**0.15**	**0.37**	**0.14**
SeedsNumber	**0.36**	**0.13**	**0.32**	**0.10**	*0.15*	*0.02*	*0.15*	*0.02*
EndFBegM	*0.33*	*0.11*	*0.33*	*0.11*	**0.35**	**0.12**	**0.32**	**0.10**
Hlp	0.32	0.10	0.35	0.12	0.31	0.10	0.35	0.12
BegFEndF	**0.30**	**0.09**	**0.28**	**0.08**	0.27	0.07	0.28	0.08
PodsNumber	**0.30**	**0.09**	**0.27**	**0.07**	**0.26**	**0.07**	**0.25**	**0.06**

**Fig. 3:**
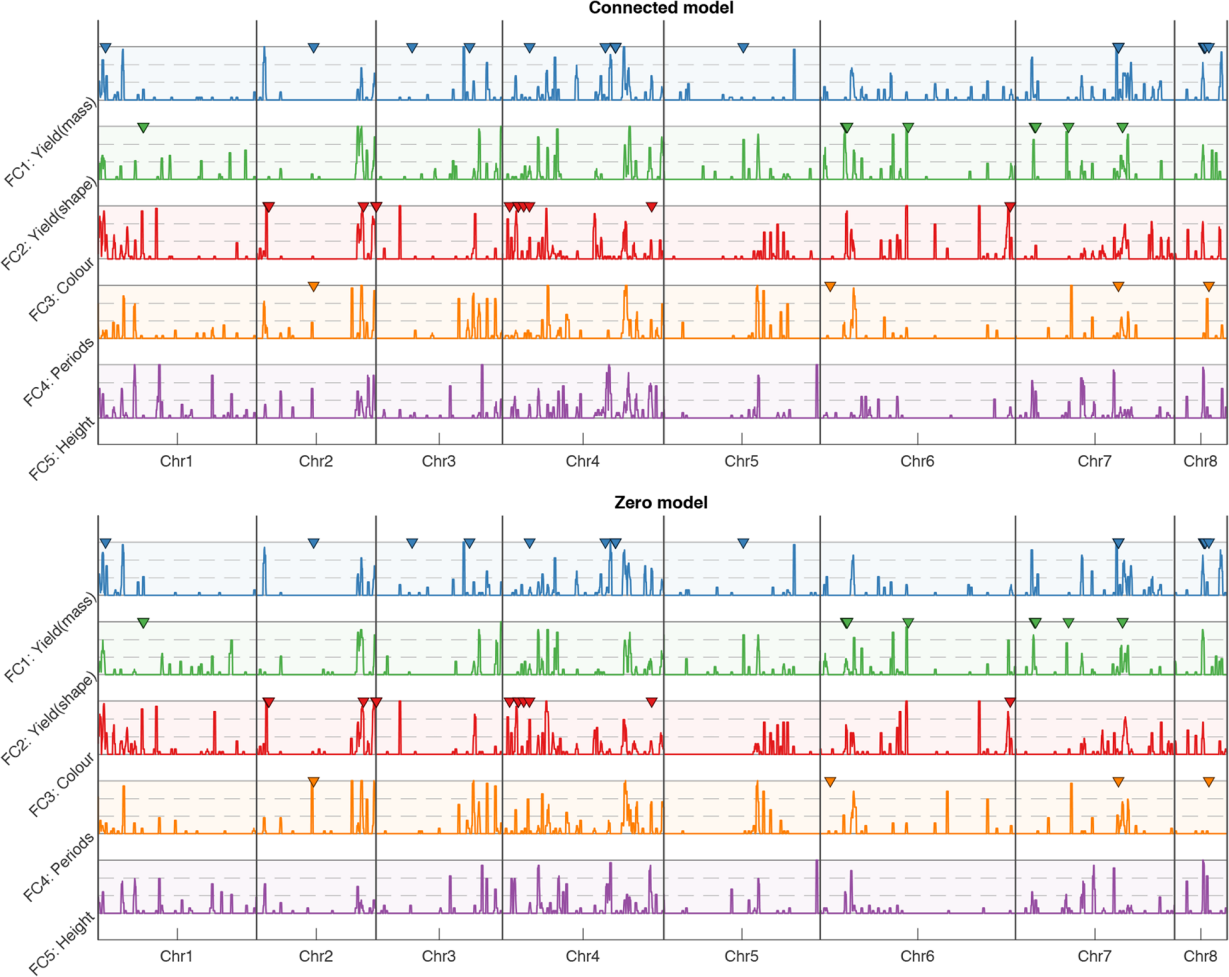
The sliding-window congruence between models obtained in 20-fold cross validation. The hight of a peak reflects the number of models having at least one SNPs within the window corresponding to the peak

Next, we analyzed positions of trait-associated SNPs on the chromosomes in both connected and zero extended model types. For each of these types, we had independently built 20 models due to the fixed 20-fold CV, and, consequently, the sets of SNPs included into the models were different. To evaluate the congruence between chromosomal positions of SNPs from different sets, we applied the sliding window technique (500 kb window size with 100 kb step) and, for each window, we counted the number of models having at least one SNP in it. We applied this technique for five subsets of SNPs separately, such that each subset was associated with a factor and its attributed phenotypes. We visualized the evaluated congruence between 20 models in Fig. 3. We found that the models agree with each other due to the significant amount of windows, where all models have SNPs. We next compared positions of peaks with GWAS-hits obtained by a single-trait, single-locus model for the chickpea dataset [38]. Utilizing the permutation test, we found that positions of the GWAS-hits and the peaks are not independent (*p*-value < 0.05) indicating that there is some concordance between our models and GWAS analysis. In Fig. 3, some GWAS hits do not have any matches with peaks, because our model does not include correlated SNPs, which naturally occur in GWAS results. Moreover, our model describes essentially more information than single-trait GWAS; therefore, some peaks do not match any GWAS hits.

## Discussion

GWAS often relies on data with a number of highly correlated phenotypic traits. Due to these correlations, significant SNPs are frequently associated with several phenotypes, i.e., they are pleiotropic. Until recently, multi-trait multi-locus models could neither distinguish SNP effects between pleiotropic and single-trait ones nor analyze a large number of traits and variants. In a SEM-based model, aggregation of pleiotropic effects into latent constructs makes it possible to distinguish SNP effects and, therefore, shed more light on mechanisms underlying associations. Large numbers of SNPs and traits in the model can lead to a parameter identification problem that, nevertheless, can be solved by applying Bayesian approach for parameter estimation.

Here we developed the mtmlSEM (multi-trait multi-locus SEM) model that estimates and evaluates casual relations between phenotypes and SNPs, reliably discriminates variant effects between single-trait and pleiotropic ones, and has good predictive ability. The developed model is a general one and can be applied to analysis of associations between variants and correlated traits in any dataset. It consists of two main steps. Firstly, the structure of the model is automatically constructed, such that correlated traits are joined into latent factors and explanatory SNPs are introduced to latent factors and phenotypic traits directly. Under this paradigm, one could consider latent factors as aggregating yet unknown biological processes that explain the SNP influence on phenotypes. At the second step, the parameter estimates are obtained with MCMC (Gibbs sampling) after the Bayesian inference of posterior distributions for parameters.

At the next step, the applicability of the mtmlSEM model was illustrated on a dataset of chickpea accessions. Many phenotypic traits in this dataset are correlated and therefore single-trait GWAS inferences can be biased. We compared four models: *zero* or *connected* means inclusion or not parameters in B, *base* or *extended* means inclusion or not parameters in K. To estimate model accuracy, we applied the 20-fold cross-validation, which led to construction of 20 different models for each model type.

After the accuracy of trait prediction was assessed, it became evident that among base models, connected ones describe the covariance structure of the data more accurately and, therefore, showed better predictive ability than the zero models. Therefore, one may conclude that joining latent factors into a structure was reasonable as all phenotypes are mutually dependent and cannot be considered as isolated blocks of traits.

In the case of extended models, the supplementary SNPs added to phenotypes described the residual variance not covered by the base models, so that the connected and zero extended models were comparable in both total numbers of SNPs and accuracy.

We next tested the utility of the models to predict associations between SNPs and phenotypes. We found that in that base and connected extended models behave similarly supporting their resemblance to one another. The associations revealed with mtmlSEM model and in standard GWAS analysis are consistent and the differences observed arise due to exclusion of correlated SNPs from the mtmlSEM models, and because mtmlSEM models consider individual and pleiotropic effects of SNPs separately. These effects could be singled out by calculating the difference between SNP effects in extended and zero models. However, the pleiotropic SNP effects are central to trait prediction in the models since the addition of SNPs to traits does not result in marked increase of prediction accuracy (see Table 2).

## Conclusions

We developed the mtmlSEM model that describes casual relations between between single-trait and pleiotropic SNPs and phenotypic traits. The particular strength of mtmlSEM model developed here is its ability to predict traits from genomic data. Notably, while the chickpea dataset used in this study is relatively small, the accuracy of the predictions for many traits was good and is comparable or even superior to the accuracy of breeding values predictions in genomic selection models. However, the applicability of mtmlSEM models in genomic selection studies requires further investigation.

## Methods

### Structural equation modeling

First proposed by S. Wright [26] for path analysis, SEM is defined today as a diverse set of tools and approaches covering regression models, path analysis and confirmatory factor analysis. The first SEM model was LISREL, and it has two distinct parts: structural and measurement [41, 42]. The structural part of LISREL reflects the causal relationships between endogenous and exogenous latent variables; the measurement model describes how latent variables influence their manifest variables:

1 $$ \begin{array}{ll}\eta & =\mathrm{B}\eta +\varepsilon \\ {}p& =\Lambda \eta +\delta \end{array}\operatorname{} $$

where *η* is a vector of *n*_*η*_ latent factors (both exogeneous and endogenous), *p* is a vector of *n*_*p*_ observed manifest variables, Λ is a matrix of factor loadings, B is a matrix of relationships between latent factors, *ε* ∼ *N*(0, Θ_ε_) and *δ* ∼ *N*(0, Θ_δ_) are random errors, Θ_ε_ and Θ_δ_ are diagonal matrices of sizes (*n*_*η*_, *n*_*η*_) and (*n*_*p*_, *n*_*p*_), respectively.

To adapt this model for genotype-phenotype studies, we considered *p* as a vector of phenotypes, and *η* as a vector of latent variables, which describe the shared variance of genetically correlated traits. One possible interpretation of the measurement part of the model in these terms is that latent variables play the role of molecular mechanisms governing the correlation between traits. The structural part describes the interplay between these mechanisms.

To construct the mtmlSEM model, we extended the LISREL model with observed exogenous variables assuming them as SNPs. New exogenous variables influence either latent factors or phenotypes traits1 and mean pleiotropic and single-trait effects, respectively. As a result, latent variables *η* become only endogenous and the SEM model is transformed as follows:

2 $$ \begin{array}{ll}\eta & =\mathrm{B}\eta +\Pi g+\varepsilon \\ {}p& =\Lambda \eta +\mathrm{K}y+\delta \end{array}\operatorname{} $$

where *g* and *y* are variables of SNPs influencing latent factors and phenotypic traits, respectively; Π and K are matrixes of SNP influences on latent factors and phenotypes, respectively. We assumed that each column of both the Π and K matrices can contain only one cell with a parameter such that each SNP can influence only one variable. SNPs in the structural part, *g*, describe a part of phenotypic variance, which is common for several traits. However, each phenotype has its own variance, which is described by SNPs in the measurement part, *y*. If the B matrix is not zero, a pleiotropic SNP, which directly influences one latent variable and its related traits, can indirectly affect other latent variables and their traits. Therefore, in mtmlSEM model, SNPs can be subdivided into single-trait, pleiotropic and direct/indirect effects.

The Maximum likelihood method, most often used to estimate parameters in SEM model, assumes that all observed and latent variables are normally distributed. Under this assumption, the sample covariance matrix of observed variables follows the Wishart distribution with the mean equal to the model-implied covariance matrix. In our dataset, some of the phenotypic traits and all SNPs take discrete ordinal values; therefore, the ML approach cannot be applied. To consider ordinal variables as normally distributed, we substituted sample covariances between ordinal variables with polychoric correlations and between ordinal and continuous variables with polyserial correlations (see section Ordinal variables). The ML approach can be applied after this manipulation (see Additional File 3).

### Construction of measurement part

We identified latent variables influencing phenotypic traits applying factor analysis (FA). To determine the number of factors, we applied the parallel analysis [43]. Then, we performed FA and attributed a trait to a factor if the absolute value of the factor loading (i.e. standardized regression coefficient) exceeds 0.5. Factors influencing less than two phenotypes and phenotypes not attributed to the factors were filtered out. As a result, we obtained the measurement part of the model (1), which is a set of latent factors that influence the subsets of phenotypic traits:

3 $$ p=\Lambda \eta +\delta $$

where Λ is a sparse matrix. The model does not contain an intercept term because traits are standardized to have mean zero and variance one.

### Construction of structural part

In FA, factors are independent and influence all observed variables. By setting some factor loadings to zero, we probably violated the factor independency; therefore, we expect them to be non-independent. To include factor dependency into the model, we allowed factors to be in causal relationships that describe presumable common variance between them:

4 $$ \eta =\mathrm{B}\eta +\varepsilon $$

where B is the coefficient matrix for relationships between latent variables, *η*. The model does not contain an intercept term because latent variables are assumed to have mean zero. Eq. (4) together with eq. (3) form the traditional LISREL model. To obtain the positions of parameters in the B matrix, we iteratively add them one by one until a stopping criterion is met. At an iteration, we considered each pair of latent factors and examined two possible relationships within the pair: to and back links. For each causal relationship not forming a cycle in the structural part, we estimated the parameters of the corresponding LISREL model by the ML method and checked for statistical significance of all the parameters in both Λ and B matrices (*p*-value < 0.05). Next, we defined the best relationship between latent factors as having the highest likelihood value and fixed the corresponding position of a new parameter in B. The iterations continued until the log-likelihood value stops decreasing.

### SNP selection

Before SNPs were incorporated into the model, we estimated parameters for the constructed LISREL part of the model (Eq. (1)) and fixed all parameter values in B and Λ matrices. This is necessary to do as SNP addition enlarges the number of parameters that makes further ML estimation unstable. Therefore, we added SNPs to the model with fixed B and Λ matrices.

We first automatically introduced SNPs for each latent variable (vector *g* in Eq. (2)) into the model starting from the exogenous latent variables and breadth-first following the direct acyclic graph (DAG) of the structural part. Then, we performed the same automatic procedure and introduced SNPs for phenotypes (vector *y* in Eq. (2)).

Selecting a SNP for a variable, whether it is a latent factor or phenotype, consisted of three steps. At the first step, we included SNPs one by one as influencing the variable and perform the ML estimation of model parameters. The sample covariance matrix of all observed variables for both phenotypic traits and SNPs follows the Wishart distribution with the mean equal to model-implied covariance matrix (see Additional File 3). Secondly, based on the ML estimates, we calculate the Wishart density for the sample covariance matrix of phenotypes only taking as the mean parameter of the distribution the model-implied covariance of phenotypes. At the third step, we sort all SNPs according to the calculated densities and put the top SNP into the model fixing the corresponding parameter in Π or K matrix with the ML estimate. This automatic algorithm for selecting SNPs was implemented using the tools of the ***semopy*** [44] Python package.

### Ordinal variables

The estimation of parameters in the SEM model is traditionally based on the assumption that all variables, whether they are observed or latent, are normally distributed. However, in the mtmlSEM model, this assumption is inevitably violated because SNPs take only discrete values, for instance, {0, 1, 2}, in the additive model. Moreover, the ordinal scale is often used for measurements of phenotypic traits.

We considered ordinal data as coming from a hidden continuous normal distribution with a threshold specification [45] and introduced additional latent variables to the model as follows. Let $\overset{\sim }{x}$ be a latent normally distributed variable that mimics the ordinal variable *x* taking values from {*x*_1_, *x*_2_, …*x*_*n*_}. Suppose for a given data set the proportions of these values are {*f*_1_, *f*_2_, …*f*_*n*_}, respectively. Let thresholds {− ∞  = *t*_0_, *t*_1_, …*t*_*n*_ = ∞} divide the normal distribution into *n* parts corresponding to the proportions *t*_*k*_ equal to the standard normal quantile at ${\sum}_{i=1}^k{f}_i$. Although the exact continuous measurements of $\overset{\sim }{x}$ are not available, we consider that if *x* = *x*_*k*_, then ${t}_{k-1}<\overset{\sim }{x}\le {t}_k$ [45]. Thereby, for each SNP and ordinal phenotypic trait, we introduce to the model additional normally distributed latent variables.

Let the vector of phenotypes *p* be split into two parts: continuous traits, *u*, modelled as normally distributed, and discrete phenotypes, *v*, measured on an ordinal scale. For the latter, as well as for *g* and *y* variables, we apply the threshold approach described above and introduce vectors of latent variables $\overset{\sim }{v}$, $\overset{\sim }{g}$ and $\overset{\sim }{y}$, respectively. Therefore, the model (2) is transformed to

5 $$ \begin{array}{ll}\eta & =\mathrm{B}\eta +\Pi \overset{\sim }{g}+\varepsilon \\ {}\left(\begin{array}{c}u\\ {}\overset{\sim }{v}\end{array}\right)& =\Lambda \eta +\mathrm{K}\overset{\sim }{y}+\delta \end{array}\operatorname{} $$

### Bayesian estimation of model parameters

The ML method is used to estimate parameters of SEM models most of the time. However, if the number of parameters is large, as in our mtmlSEM model, this method is computationally unstable and prone to optimization failure. In contrast to the ML method, the Bayesian approach can cope with this situation taking into account prior information about parameters and maximizing the posterior distribution of parameters and latent variables. We considered values in the B, Λ, Π, K matrices that were fixed during model construction as prior information and performed the Bayesian inference to obtain the posterior distributions for all parameters (denote set of all parameters as *ϕ* = {B, Λ, Π, K, Θ_ε_, Θ_δ_ }) and latent variables ($\eta, \overset{\sim }{v},\overset{\sim }{g},\overset{\sim }{y}$) (see Additional File 4). As a result, we were able to generate posterior distributions of parameters by the Gibbs sampler, a Markov chain Monte Carlo algorithm. We initiated each chain with random values, and, at each iteration of the sampler, we draw

1. datasets for $\overset{\sim }{v}$, $\overset{\sim }{g}$ and $\overset{\sim }{y}$ from truncated normal distributions, independently of *ϕ*;
2. datasets for *η* from the multivariate normal distribution conditional on *ϕ*;
3. diagonal values in Θ_*ε*_ from the inverse gamma distribution conditional on *ϕ*;
4. values in rows of the block matrix [B, Π] from multivariate normal distributions conditional on *ϕ*;
5. diagonal values in Θ_*δ*_ from the inverse gamma distribution conditional on *ϕ*;
6. values in rows of the block matrix [Λ, K] from multivariate normal distributions conditional on *ϕ*.

To get parameter estimates, we performed Gibbs sampling on 5 chains of length 2000, checked convergence indicators (Gelnman-Rubin diagnostics and the effective sample size), and calculated the parameter estimates.

### The chickpea dataset

**Fig. 4:**
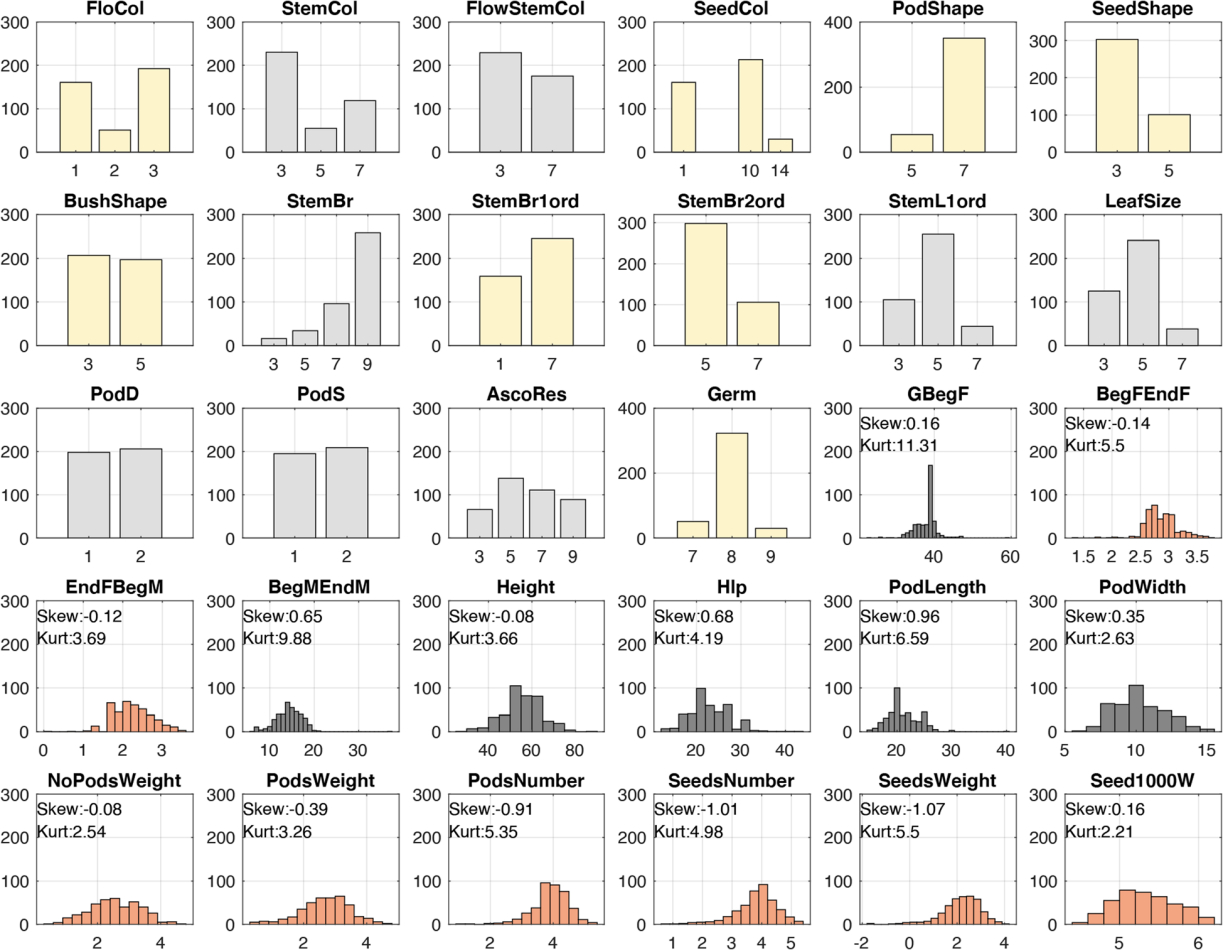
Distributions of the data after preparation. Grey-coloured traits were not transformed. Yellow-coloured traits are categorial traits that were transformed; orange-coloured traits are non-categorial and were log-transformed

The chickpea dataset (*Cicer arietinum L.*) consists of 404 accessions from the Vavilov Institute of Plant Genetic Resources (VIR) seed bank. In 2017, these accessions were phenotyped for 30 phenological, morphological, agronomical, and biological traits. Some of these traits are categorical and others are quantitative. Phenotype abbreviations and units of measurement are in Additional File 2. Genotyping by sequencing (GBS) of chickpea accessions identified 56,855 segregating single nucleotide polymorphisms (SNPs). These SNPs were further filtered to meet requirements for minor allele frequency (MAF) > 3% and genotype call-rate > 90%. 2579 SNPs in 404 accessions passed all filtering criteria and were retained for further analysis. The phenotype data were further transformed in two ways. Firstly, for some categorial traits, we merged categories to make them more distinct (Additional File 2). Secondly, several quantitative traits were log-transformed to satisfy the assumption of normality (Fig. 4). All quantitative traits were further centered and scaled by calculation of z-score.

### Test for predictive ability

The model was validated by 20-fold cross-validation. We randomly partitioned the dataset into 20 training (about 380 samples) and test (20 samples) sets and fixed the splits. For each training set, we independently constructed an mtmlSEM model and obtained parameter estimates after Gibbs sampling on 5 chains taking these parameters to predict values of phenotypic traits in the corresponding test set. The prediction accuracy was estimated by calculating the Pearson correlation between observed and predicted values across all test sets, the coefficient of determination and normalized rooted mean square error (Additional File 5).

## Supplementary information

**Additional File 1.** Absolute values of correlations between phenotypic traits.

**Additional File 2.** Description of phenotypic trait.

**Additional File 3.** Maximum Likelihood estimates.

**Additional File 4.** Bayesian inference and Gibbs sampling.

**Additional File 5.** Root mean square error.

## Data Availability

The datasets analyzed and the scripts during the current study are available in the [GitHub] repository, https://github.com/iganna/mtmlSEM.git All figures were created by A.Igolkina.

## Abbreviations

SEM: Structural equation modeling
mtmlSEM: Multi-train multi-locus SEM
GWAS: Genome-wide association studies
PCA: Principal component analysis
FA: Factor analysis
