# Supplementary figures and images for: Multi-trait multi-locus SEM model discriminates SNPs of different effects
Source: BMC Genomics. 2020 Jul 28;21(Suppl 8):490. doi: 10.1186/s12864-020-06833-2 (PMC7385891; doi:10.1186/s12864-020-06833-2)

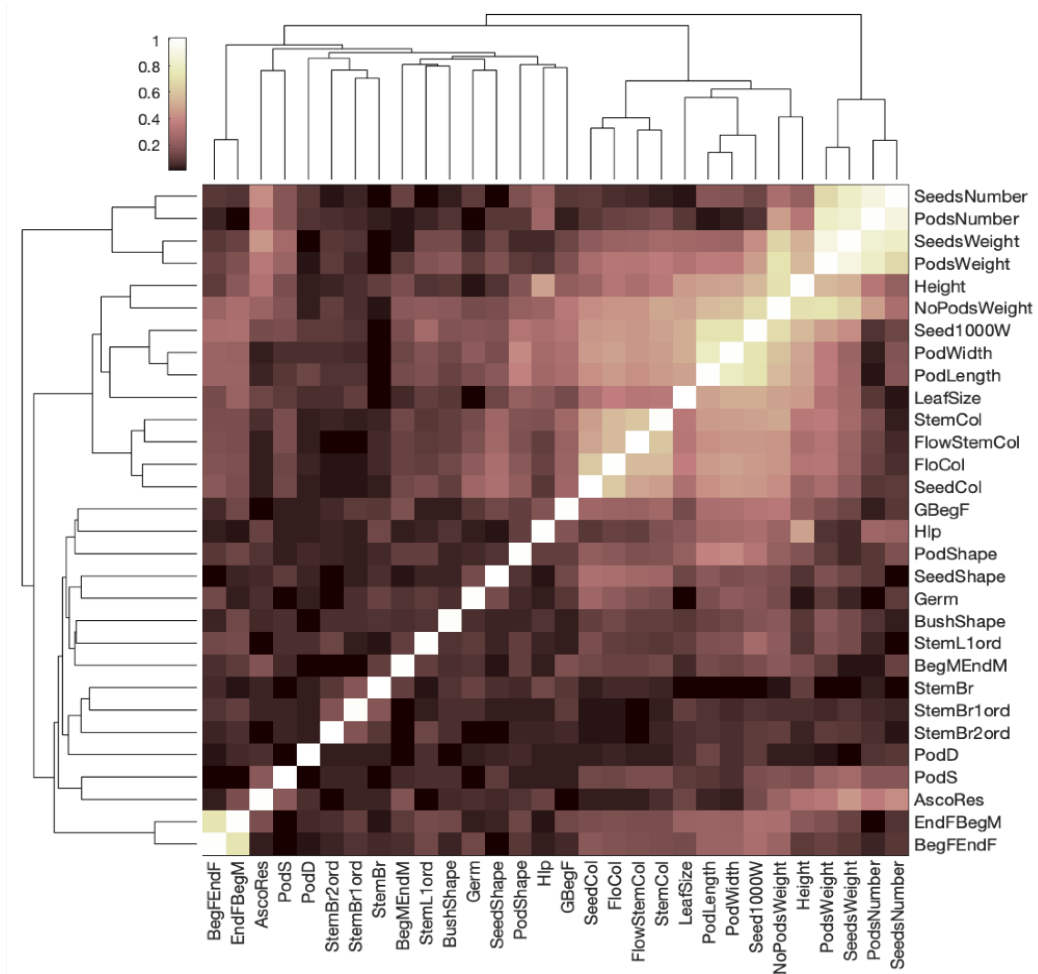

**Fig. S1.** Absolute values of correlations between phenotypic traits

Supplement: Supplementary file 1 — Additional File 1. Absolute values of correlations between phenotypic traits. [file 12864_2020_6833_MOESM1_ESM.pdf]
