# Supplementary material for: Multi-trait multi-locus SEM model discriminates SNPs of different effects
Source: BMC Genomics. 2020 Jul 28;21(Suppl 8):490. doi: 10.1186/s12864-020-06833-2 (PMC7385891; doi:10.1186/s12864-020-06833-2)
Supplement: Supplementary file 3 — Additional File 3. Maximum Likelihood estimates. [file 12864_2020_6833_MOESM3_ESM.pdf]

ML estimates

Let consider the SEM model with latent variables η, observed variables g and y and manifest variables p as following:

$$\begin{aligned} \eta &= \mathrm{B}\eta + \Pi g + \varepsilon, \\ p &= \Lambda\eta + \mathrm{K}y + \delta, \end{aligned}$$

where B, Π, Λ and K are matrices of linear relationships between variables, ε and δ are normally distributed random errors with zero means and diagonal covariance matrices Θ_ε and Θ_δ, respectively. The total set of parameters, θ, consists of B, Π, Λ, K, Θ_ε and Θ_δ. In the Maximum likelihood (ML) approach for parameter estimation, it should be assumed that all variables are normally distributed. Under this assumption, the sample covariance matrix, S, follows the Wishart distribution: $S \sim \mathcal{W}(\Sigma/n, n)$, where n is the sample size and Σ is model-implied covariance matrix. Σ contains variances and covariances for the whole set of observed variables, p, g and y , and, therefore, it has the block representation:

$$\Sigma = \begin{bmatrix} \Sigma_{\mathrm{p,p}} & \Sigma_{\mathrm{p,g}} & \Sigma_{\mathrm{p,y}} \\ \Sigma_{\mathrm{p,g}}^{\mathrm{T}} & \Psi_{\mathrm{g}} & \Psi_{\mathrm{g,y}} \\ \Sigma_{\mathrm{p,y}}^{\mathrm{T}} & \Psi_{\mathrm{g,y}}^{\mathrm{T}} & \Psi_{\mathrm{y}} \end{bmatrix},$$

$$\Sigma_{\mathrm{p,p}} = \Lambda\mathrm{C}\Pi\Psi_{\mathrm{g}}\Pi^{\mathrm{T}}\mathrm{C}^{\mathrm{T}}\Lambda^{\mathrm{T}} + \Lambda\mathrm{C}\Theta_\varepsilon\mathrm{C}^{\mathrm{T}}\Lambda^{\mathrm{T}} + \mathrm{K}\Psi_{\mathrm{y}}\mathrm{K}^{\mathrm{T}} + \Theta_\delta,$$

$$\Sigma_{\mathrm{p,g}} = \Lambda\mathrm{C}\Pi\Psi_{\mathrm{g}}\Pi^{\mathrm{T}}\mathrm{C}^{\mathrm{T}}\Lambda^{\mathrm{T}},$$

$$\Sigma_{\mathrm{p,y}} = \mathrm{K}\Psi_{\mathrm{y}}\mathrm{K}^{\mathrm{T}},$$

where $\mathrm{C} = (\mathrm{I} - \mathrm{B})^{-1}$; Ψ_g, $\Psi_{g,y}$, Ψ_y are sample covariance matrices for exogenous observed variables. ML estimates of parameters are those that result in the highest value to the Wishart density, or five the minimum value of the log likelihood ratio - a natural logarithm of the ratio of Wishart densities for a given model and for a perfectly fitting mode ($\Sigma = S$). As a result, for ML estimator, the objective function to minimise is:

$$F_M LW(\theta) = \mathrm{tr}[S\Sigma(\theta)^{-1}] + \ln|\Sigma(\theta)|.$$

The ML estimation of model parameters was performed in **semopy** Python package.
