# Supplementary material for: Multi-trait multi-locus SEM model discriminates SNPs of different effects
Source: BMC Genomics. 2020 Jul 28;21(Suppl 8):490. doi: 10.1186/s12864-020-06833-2 (PMC7385891; doi:10.1186/s12864-020-06833-2)
Supplement: Supplementary file 4 — Additional File 4. Bayesian inference and Gibbs sampling. [file 12864_2020_6833_MOESM4_ESM.pdf]

Bayesian inference and Gibbs sampling.

The final representation of our genotype-phenotype SEM model is:

$$\eta = \mathrm{B}\eta + \Pi\,\tilde{g} + \varepsilon$$

$$\binom{u}{\tilde{v}} = \Lambda\eta + \mathrm{K}\tilde{y} + \delta,$$

where latent variables $\tilde{g}$, $\tilde{y}$ and $\tilde{v}$ mimic ordinal observed variables g, y and v. Let the size of the dataset be n and it containes matrices of observations for g, y and v variables: G of size $(n_g \times n)$, Y of size $(n_y \times n)$ and V of size $(n_v \times n)$, respectively. At each iteration of an MCMC method, we generate matrices of observation for respective latent variables - $\tilde{G}$ for $\tilde{g}$, $\tilde{Y}$ for$\tilde{y}$ and $\tilde{V}$ for $\tilde{v}$ - by the same way. For example, the element in $\tilde{G}$ at i-th row and j-column is randomly drawn from standard normal distribution truncated to the support $s(G,i,j) = (l(G,i,j), r(G,i,j)]$:

$$l(G,i,j) = Q\left(\frac{1}{n}\sum_{k=1}^{n}[G_{ik} < G_{ij}]\right), r(G,i,j) = Q\left(\frac{1}{n}\sum_{k=1}^{n}[G_{ik} \leq G_{ij}]\right),$$

where $Q(\cdot)$ is the quantile function associated with the standard normal distribution, $[\dots]$ are the Iverson brackets (Knuth 1992). Thereby, we generated elements in $\tilde{G}$, $\tilde{Y}$ and $\tilde{V}$ form truncated normal distributions independently from other parameters and variables in the model:

(i) $\forall(i,j) \in \{1..n_g\} \times \{1..n\}$: generate $\tilde{G}_{ij}$ from $\mathcal{trN}(0,1,s(G,i,j))$;
(ii) $\forall(i,j) \in \{1..n_y\} \times \{1..n\}$: generate $\tilde{Y}_{ij}$ from $\mathcal{trN}(0,1,s(Y,i,j))$;
(iii) $\forall(i,j) \in \{1..n_v\} \times \{1..n\}$: generate $\tilde{V}_{ij}$ from $\mathcal{trN}(0,1,s(V,i,j))$.

To obtain the posterior distribution for latent variables η, we applied the approach proposed in (Lee 2007) at p.83. Then, for j-th sample, we denote the values of latent variables η as $H_{.j}$ and draw it from multivariate normal distribution:

(iv) $\forall j \in \{1..n\}$: generate $H_{.j}$ from $\mathcal{mvN}((\Sigma_\eta^{-1} + \Lambda^\top\Theta_\delta^{-1}\Lambda)\Lambda^\top\Theta_\delta^{-1}\,\tilde{P}_{.j}\,, (\Sigma_\eta^{-1} + \Lambda^\top\Theta_\delta^{-1}\Lambda))$, where $\Sigma_\eta = \mathrm{C}(\Pi\Pi^\top + \Theta_\varepsilon)\mathrm{C}^\top$, $\mathrm{C} = (\mathrm{I} - \mathrm{B})^{-1}$ and $\tilde{P}_{.j} = (U_{.j}^\top, \tilde{V}_{.j}^\top)^\top - \mathrm{K}\,\tilde{G}_{.j}$

Parameters in the structural part

We performed the Bayesian inference of posterior distributions for parameters in the structural part $(\mathrm{B}, \Pi, \Theta_\varepsilon)$ similarly to (Lee 2007) at p.84. Let consider an equation in the structural part corresponding to i-th latent variable η_i:

$$\eta_i = \Gamma_i\omega_i + \varepsilon_i,$$

where Γ_i is a set of parameters in i-th rows of B and Π; ω_i contains subsets of η and $\tilde{g}$ corresponding to positions of parameters in i-th rows of B and Π; ε_i is i-th component in the vector of random errors and it is normally distributed with zero mean and variance equal to $\Theta_{\varepsilon i}$. We set the inverse Gamma and multivariate normal prior distributions for $\Theta_{\varepsilon i}$ and Γ_i respectively:

$$\Theta_{\varepsilon i} \sim \mathcal{IG}(\alpha_{0\varepsilon i}, \beta_{0\varepsilon i}),$$

$$\Gamma_i \sim mv\mathcal{N}(\mathrm{F}_{0i}, \Theta_{\varepsilon i}\Phi_{0i}),$$

where F_{0i} is the ML estimate for Γ_i, Φ_{0i} is the Identity matrix; parameters for inverse Gamma distribution are taken as in (Lee 2007) at p.76: $\alpha_{0\varepsilon i} = 9, \beta_{0\varepsilon i} = 4$. Let the dataset for ω_i be the matrix Ω_i of size $\left(n_{\omega_i} \times n\right)$; the dataset for η_i be the horizontal vector H_i of length n. During the Bayesian inference of posterior distributions, we obtained the following parameters of posterior distributions:

$$\Phi_i = (\Phi_{0i}^{-1} + \Omega_i\Omega_i^{\mathrm{T}})^{-1},$$

$$\mathrm{F}_i = \Phi_i[\Phi_{0i}^{-1}\mathrm{F}_{0i} + \Omega_i H_i^{\mathrm{T}}],$$

$$\alpha_{\varepsilon i} = \alpha_{0\varepsilon i} + n/2,$$

$$\beta_{\varepsilon i} = \beta_{0\varepsilon i} + \frac{1}{2}[H_i H_i^{\mathrm{T}} + \mathrm{F}_{0i}^{\mathrm{T}}\Phi_{0i}^{-1}\mathrm{F}_{0i} - \mathrm{F}_i^{\mathrm{T}}\Phi_i^{-1}\mathrm{F}_i].$$

Then, values of Γ_i and $\Theta_{\varepsilon i}$are drawn in the following order:

(v) generate $\Theta_{\varepsilon i}$ form $\mathcal{IG}(\alpha_{\varepsilon i}, \beta_{\varepsilon i})$,
(vi) generate Γ_i form $mv\mathcal{N}(\mathrm{F}_i, \Theta_{\varepsilon i}\Phi_i)$.

Parameters in the measurement part

As for the structural part, we performed the Bayesian inference of posterior distributions for parameters in the measurement part $(\Lambda, \mathrm{K}, \Theta_\delta)$ similarly to (Lee 2007) at p.84. Let consider an equation in the measurement part corresponding to i-th phenotype p_i:

$$p_i = \mathrm{A}_i w_i + \delta_i,$$

where A_i is a set of parameters in i-th rows of Λ and K; w_i contains subsets of η and $\tilde{y}$ corresponding to positions of parameters in i-th rows of Λ and K; δ_i is i-th component in the vector of random errors and it is normally distributed with zero mean and variance equal to $\Theta_{\delta i}$. We set the inverse Gamma and multivariate normal prior distributions for $\Theta_{\delta i}$ and A_i respectively

$$\Theta_{\delta i} \sim \mathcal{IG}(\alpha_{0\delta i}, \beta_{0\delta i}),$$

$$\mathrm{A}_i \sim mv\mathcal{N}(\mathrm{D}_{0i}, \Theta_{\delta i}\Psi_{0i})$$

where D_{0i} is the ML estimate for A_i, Ψ_{0i} is the Identity matrix; parameters for inverse Gamma distribution are taken as in (Lee 2007) at p.76: $\alpha_{0\delta i} = 9, \beta_{0\delta i} = 4$. Let the dataset for w_i be the matrix W_i of size $\left(n_{w_i} \times n\right)$; the dataset for p_i be the horizontal vector P_i of length n. During the Bayesian inference of posterior distributions, we obtained the following parameters of posterior distributions:

$$\Psi_i = (\Psi_{0i}^{-1} + W_i W_i^{\intercal})^{-1},$$

$$\mathrm{D}_i = \Psi_i[\Psi_{0i}^{-1}\mathrm{D}_{0i} + W_i P_i^{\intercal}],$$

$$\alpha_{\delta i} = \alpha_{0\delta i} + n/2,$$

$$\beta_{\delta i} = \beta_{0\delta i} + \frac{1}{2}[P_i P_i^{\intercal} + \mathrm{D}_{0i}^{\intercal}\Psi_{0i}^{-1}\mathrm{D}_{0i} - \mathrm{D}_i^{\intercal}\Psi_i^{-1}\mathrm{D}_i].$$

Then, values of A_i and $\Theta_{\delta i}$are drawn in the following order:

(vii) generate $\Theta_{\delta i}$ form $\mathcal{IG}(\alpha_{\delta i}, \beta_{\delta i})$,
(viii) generate A_i form $\mathcal{mvN}(\mathrm{D}_i, \Theta_{\delta i}\Psi_i)$.

Scripts are available at https://github.com/iganna/mtmlsem.
