# Supplementary material for: Multi-trait multi-locus SEM model discriminates SNPs of different effects
Source: BMC Genomics. 2020 Jul 28;21(Suppl 8):490. doi: 10.1186/s12864-020-06833-2 (PMC7385891; doi:10.1186/s12864-020-06833-2)
Supplement: Supplementary file 5 — Additional File 5. Root mean square error. [file 12864_2020_6833_MOESM5_ESM.pdf]

Table.

Values of root mean square error, normalized to the mean value of phenotypic trait.

	Zero extended	Connected extended	Zero base	Connected base
BegFEndF	0.10	0.10	0.10	0.10
EndFBegM	0.21	0.21	0.21	0.20
Height	0.13	0.13	0.14	0.14
Hlp	0.19	0.20	0.19	0.19
NoPodsWeight	0.23	0.23	0.26	0.25
PodLength	0.10	0.10	0.11	0.11
PodWidth	0.13	0.14	0.13	0.14
PodsNumber	0.14	0.14	0.14	0.14
PodsWeight	0.26	0.26	0.26	0.26
Seed1000W	0.05	0.05	0.05	0.05
SeedsNumber	0.18	0.17	0.18	0.18
SeedsWeight	0.36	0.35	0.35	0.35
Median value	0.16	0.16	0.16	0.16
